# Acute Simple Elbow Dislocations in the United States: An Epidemiological Analysis of Trends Including COVID-19

**DOI:** 10.7759/cureus.57781

**Published:** 2024-04-07

**Authors:** Michael D Baird, Alexis B Sandler, John P Scanaliato, Annette H Yoon, Morgan R Askew, Kyle J Klahs, John C Dunn, Nata Parnes

**Affiliations:** 1 Orthopaedic Surgery, Walter Reed National Military Medical Center, Bethesda, USA; 2 Orthopaedic Surgery, Texas Tech University Health Sciences Center El Paso, El Paso, USA; 3 Orthopaedic Surgery, Midwest Orthopaedics at Rush University, Chicago, USA; 4 Medical School, Brown University, Providence, USA; 5 Shoulder and Elbow Surgery, Carthage Area Hospital, Carthage, USA

**Keywords:** ulnohumeral joint, epidemiology, covid-19, dislocations, elbow

## Abstract

Background

Elbows are one of the most frequently dislocated large joints; however, there is limited epidemiological data, especially during the coronavirus disease 2019 (COVID-19) pandemic. This study characterizes elbow dislocations presenting to Emergency Departments (EDs) over the last decade.

Methods

This study is a cross-sectional, descriptive, epidemiologic analysis of isolated elbow dislocations presenting to EDs from 2011-2020 using the National Electronic Injury Surveillance System (NEISS) database. Patients under 10, those with radial head subluxation, and those with complex fractures were excluded. Data on incidence ratios, patient demographics, mechanisms of injury, and incident locales were analyzed.

Results

Approximately 83,996 simple, primary elbow dislocations occurred from 2011-2020 (n=2,328), generating an incidence of 2.98/100,000 person-years. Incidence was higher among males (3.26 versus 2.69/100,000 person-years). Dislocations peaked in patients aged 10-19, with higher rates in males (11.12 versus 5.31/100,000 person-years; injury rate ratio 2.09, CI=2.05-2.14, p<0.001). Rates of elbow dislocations decreased with age in males (age 20-29=11.12, age >80=0.63/100,000) but increased in females over 40 (age 40-49=1.59, age 70-79=2.83/100,000). Athletic activities accounted for 55% of dislocations (n=45,902), with 15% from football and 14% from wrestling. The fewest annual dislocations occurred during COVID-19 (n=6440). Injuries occurring at schools and during contact and indoor sports decreased, while those from soccer increased.

Conclusions

Elbow dislocations are common, with trends of decreasing incidence with age among men and increasing incidence in women over 40. COVID-19 impacted sports-related and epidemiologic injury patterns. Ultimately, understanding population-level risks for elbow dislocations enables orthopaedic surgeons to predict injury trends and conceive educational preventative measures.

## Introduction

Elbow dislocations are a pervasive traumatic injury: the elbow is the second most commonly dislocated major joint in adults and, while elbow dislocations are seen across patients of all ages, they disproportionally affect younger, active patients [1-3]. Despite being an inherently stable joint due to its three points of articulation and extensive soft tissue stabilization, the elbow is at risk for dislocation in extension [4]. In the extended position, an axial force levers the ulna from the trochlear articulation and a series of rotational forces can disrupt the medial and lateral ulnar collateral ligaments and then anterior capsule, resulting in an unstable joint primed for dislocation [2,4-6]. Elbow dislocations can be further subclassified into simple and complex injuries, with simple dislocations defined as isolated soft tissue disruptions and complex dislocations involving osseous sequelae.

In the United States (US), a staggering 45% of elbow dislocations are attributed to sporting activities [2]. Football players are at an especially increased risk. Only a few epidemiologic studies since 1980 have been published, leaving gaps in the population-wide understanding of the injury. A 2021 review of 15 seasons of injury data from the National Football League Injury Surveillance System (NFLISS) demonstrated an incidence of 0.26 elbow dislocations per 10,000 ‘athlete exposures,’ which they defined as each time an athlete participates in a single practice or a game [7].

The purposes of this study are twofold: first, to describe the current epidemiological and demographic trends related to simple elbow dislocations without associated fractures across the US over the last decade and second, to better characterize how trends in these often sports-related injuries were affected by the ongoing COVID-19 pandemic. We hypothesized that elbow dislocations would occur most frequently during athletic events and that incidence would decrease inversely with patient age.

## Materials and methods

Database

We performed a cross-sectional, descriptive, epidemiologic study with data obtained from the US Consumer Product Safety Commission’s (CSPC) National Electronic Injury Surveillance System (NEISS) in compliance with RECORD guidelines. The NEISS database, a well-established source for characterizing orthopaedic injuries, is a repository of injury-related data collected as a probability sample from approximately 100 Emergency Departments (EDs) across the US that represent geographic and size-based distributions of hospitals across the country [8-12]. NEISS data is obtained by a hospital-specific coordinator who reviews and codes all ED visits based on available clinical information and telephone follow-up if necessary [13]. Based on standardized demographics and hospital-specific descriptive information, NEISS data can be weighted to calculate how the encounter is representative of the national populous.

Search query

The NEISS database search included the phrase "elbow dislocation" in the 10-year period from 2011 to 2020. Patients under the age of 10 were excluded due to the high incidence of radial head subluxation rather than true elbow dislocation in this age range. In the remaining cases, qualitative descriptive data were reviewed to ensure all cases denoted as radial head subluxations, complex elbow dislocations, and recurrent dislocations were removed.

Data collection

Deidentified data were obtained from the NEISS database. Incidence data included rates of injuries from the NEISS data as well as estimated national incidences based on census data. Demographic data included patient age and sex. Mechanism data were divided into sport-related and non-sport-related mechanisms and included the specific activities associated with injury as specified in NEISS. Further interrogation of the change in injury pattern among age- and sex-stratified patients with sports versus non-sport mechanisms was performed by subdividing into 30-year groups of 10-39, 40-69, and 70-99 based on the available census data stratifications. Location data included the location at which the incident resulting in the index injury occurred.

Statistical analysis

Raw and weighted descriptive statistics were assessed for all cases meeting inclusion criteria. Recent US census data from 2020 were used to estimate national incidences specific to age and sex population distributions. Incidence rates per 100,000 persons at risk and 95% confidence intervals (CIs) were calculated in addition to weighted case incidences for select injuries occurring in the study period. Mid-p two-sided p-values were calculated for all incidence rate ratios and Poisson approximations were utilized to construct incidence rate CIs. Chi-squared tests were used to compare dislocation rates in comparable patient cohorts and t-tests to compare trends in dislocation rates over time. All analyses were conducted in Stata 17 software (StataCorp., College Station, TX, USA) [14].

## Results

Incidence

**Figure 1:**
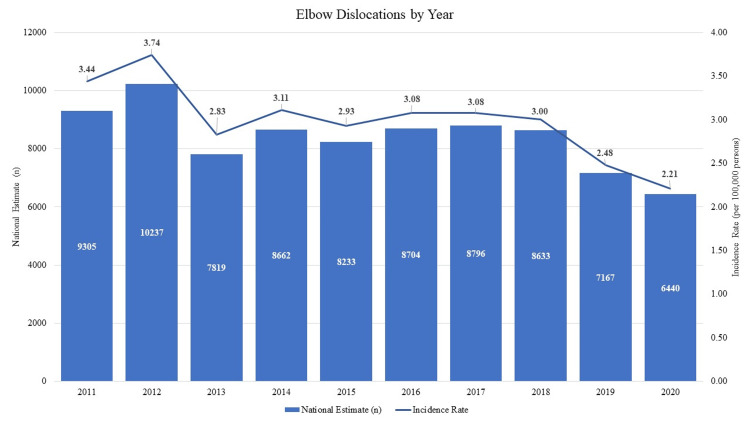
Elbow Dislocations by Year

An estimated 83,996 primary elbow dislocations without associated fractures occurred nationwide over the 10-year study period from 2011 and 2020 as calculated from the 2,328 cases included in NEISS database. A majority (estimated n = 46,855, 56%) of patients were male and between ages 20 - 29 (estimated n = 14,978, 18%). The overall incidence of isolated elbow dislocations among people 10 years and older was 2.98 dislocations per 100,000 person-years with a cumulative incidence of 44.4 dislocations per 100,000 people (CI = 43.2 - 45.6) over the study period. In general, the rate of elbow dislocations has significantly decreased over the last decade (p<.001), with the lowest injury rates noted in 2020 (Figure 1).

Demographics

**Figure 2:**
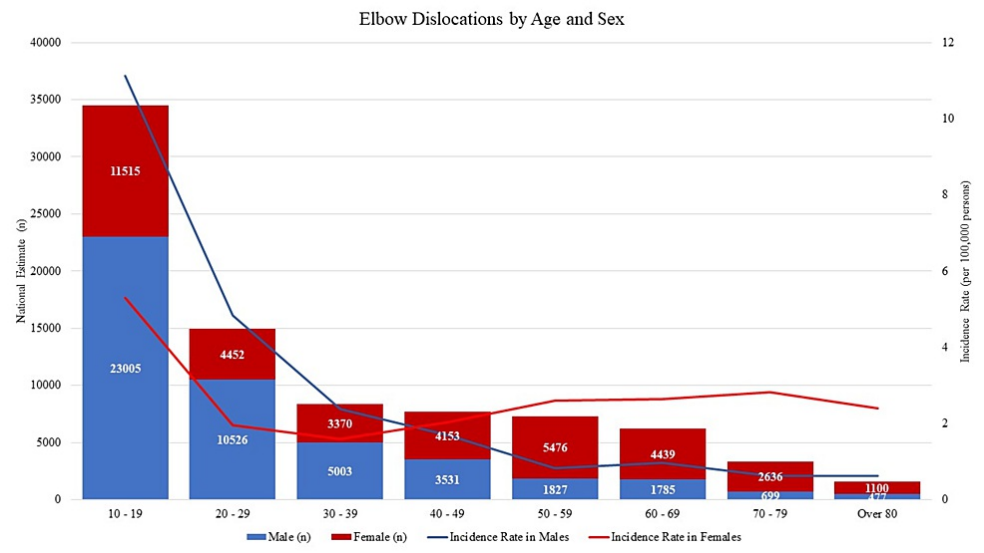
Elbow Dislocations by Age and Sex

The overall incidence rate of all males was 3.26 dislocations per 100,000 person-years while that of females was 2.69 dislocations per 100,000 person-years. When stratifying patient age by decade, the highest number of dislocations occurred in patients aged 10 - 19, with an estimated 23,005 nationally in males (27.39%, NEISS data n = 712) and 11,514 nationally in females (13.71%, NEISS data n = 401) (Figure 2). In these patients, the incidence of injury in males was significantly higher than that in females: elbow dislocations occurred in male patients at a rate of 11.12 injuries per person year versus in female patients at a rate of 5.31 injuries per person-year, resulting in an injury rate ratio of 2.09 (CI = 2.05 - 2.14, p<0.001). The incidence rate ratio between males and females peaked in patients ages 20 - 29 with 2.46 times more injuries in males (4.83 for males versus 1.96 for females, CI = 2.38 - 2.55, p<0.001). Higher incidence rates among male patients continued up until age 40, after which female patients presented with incidence rates of 2.03 (CI = 1.96 - 2.09) versus male rates of 1.69 (CI = 1.63 - 2.75) in patients aged 40 - 49 (incident rate ratio = 0.83, CI = 0.80 - 0.87, p<.001) and the even more stark difference of 2.64 (CI = 2.56 - 2.72) on females versus 0.96 (CI = 0.92 - 1.01) in males over 60-69 (incident rate ratio = 0.36, CI = 0.34 - 0.38, p<.001).

When stratified by age group, the rate of patients with elbow dislocations decreased with every 10-year increase in age (p<.001). When further grouped by sex, incidence among male patients fits this trend, decreasing from 11.1 per 100,000 in patients aged 20 - 29 to 0.63 per 100,000 in those over 80. However, this drastic rate of decreasing incidence is not replicated among female patients, whose dislocation rates decrease from 5.31 per 100,000 in patients aged 20 - 29 to 1.59 in patients 40 - 49 before increasing to 2.83 dislocations per 100,000 in those aged 70 - 79.

Mechanism of injury

**Figure 3:**
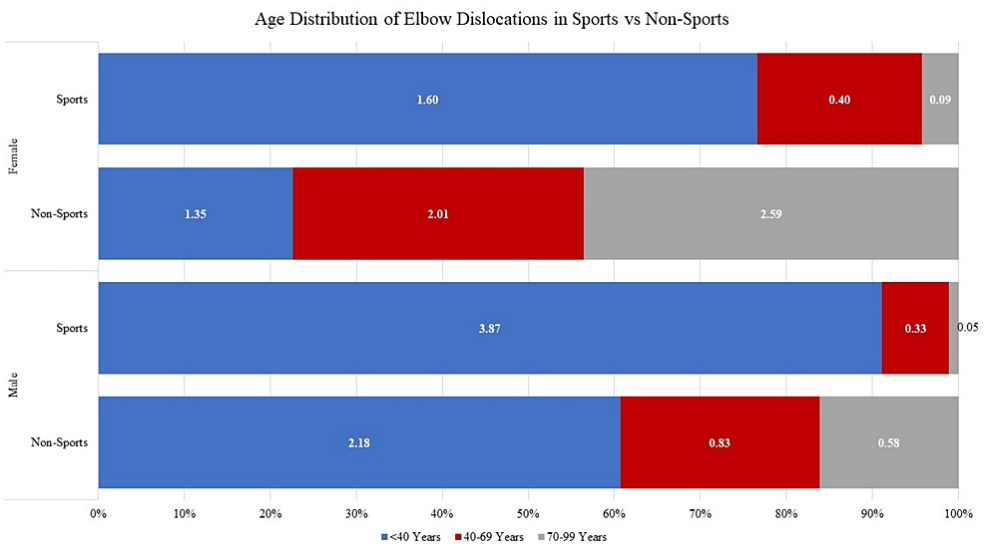
Age Distribution of Elbow Dislocations in Sports vs Non-Sports

Athletic activities were responsible for an estimated 45,902 dislocations nationally during the study period (54.7%, NEISS data n = 1,363) with a cumulative rate of 1.41 dislocations per 100,000 people. When compared to non-sports-related mechanisms in stratified age groups, sports-related mechanisms in males were highest in the under 40 age category and declined over time (3.87 sports versus 2.18 non-sports dislocations per 100,000 in patients under 40; 0.33 versus 0.83 in patients 40 - 69; and 0.05 to 0.58 in patients 70 and over). Among females, a similar rate of decline was observed among the rate of those injured in sports; however, injuries from non-sports related mechanisms actually increased with increasing age category (1.60 sports versus 1.35 non-sports dislocations per 100,000 in patients under 40; 0.40 versus 2.01 in patients 40 - 69; and 0.09 to 2.59 in patients 70 and over) (Figure 3).

**Figure 4:**
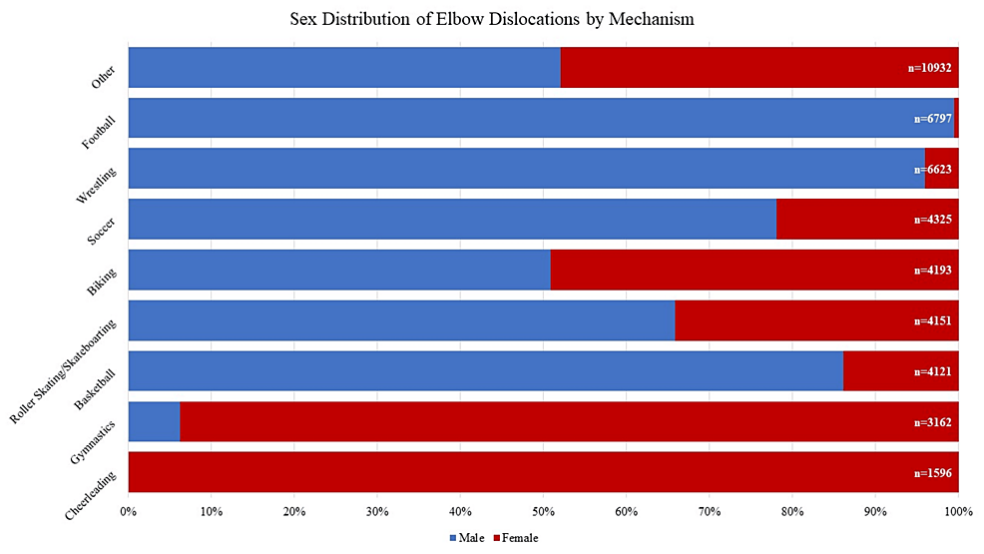
Sex Distribution of Elbow Dislocation by Mechanism

Football and wrestling represented the sports with the most common incidence of isolated elbow dislocation, accounting for 15% (NEISS data n = 241; national weighted estimate n = 6797) and 14% (NEISS data n = 189; national weighted estimate n = 6623) of cumulative dislocations, respectively (Table 1, Figure 4). Soccer, biking, roller skating/skateboarding, and basketball each accounted for 9% of the total weighted dislocations in athletic activities and gymnastics for 7%, with none of the remaining athletic activities comprising more than 3% of other sports-related mechanisms.

**Table 1:** Elbow Dislocations and Implicated Sports

Sport/Activity	National Estimate (n)	Sex		% of Sports-Related Dislocations
		Male	Female	
Football	6797	6763	34	14.8%
Wrestling	6623	6353	271	14.4%
Soccer	4325	3376	949	9.4%
Biking	4193	2133	2061	9.1%
Roller Skating/Skateboarding	4151	2736	1415	9.0%
Basketball	4122	3549	573	9.0%
Gymnastics	3163	199	2964	6.9%
Cheerleading	1596	0	1596	3.5%
Trampoline	1517	709	808	3.3%
Ski/Snowboard	1370	847	523	3.0%
Baseball/Softball	1297	937	359	2.8%
Horses	1241	418	823	2.7%
Rock Climbing	1139	543	597	2.5%
Martial Arts	806	750	56	1.8%
Dancing	596	47	549	1.3%
Swimming	392	179	213	0.85%
Volleyball	372	47	325	0.81%
Track	323	38	285	0.70%
Mountain Climbing	300	216	84	0.65%
Tennis	250	217	34	0.55%
Surfing	231	80	150	0.50%
Weightlifting	219	164	55	0.48%
Lacrosse	214	118	96	0.47%
Water Skiing	160	91	68	0.35%
Hockey	125	109	16	0.27%
Skating	103	32	70	0.22%
Bowling	91	15	76	0.20%
Handball	74	74	0	0.16%
Golf	64	14	49	0.14%
Ice Skating	17	17	0	0.04%
Squash	16	16	0	0.04%
Boxing	16	16	0	0.03%
Total	45902	30804	15098	100%

Incident locale

The greatest proportion of elbow dislocations occurred at places of recreation or sport (30%, NEISS data n = 823) followed by the home (25%, NEISS data n = 509), and school (9%, NEISS data n = 201).

COVID-19 pandemic

The fewest cumulative dislocations (NEISS data n = 192, national estimate n = 6440) occurred in 2020, despite the largest US population numbers at this time. The mechanisms of injury also changed in 2020: the number of elbow dislocations from contact sports such as football and wrestling decreased by 41% and 64%, respectively, while the number of dislocations from indoor sports such as basketball and gymnastics also decreased by 48% and 62%, respectively, when compared to the mean number of injuries from 2011 - 2019 (Table 2). Interestingly, dislocations during soccer increased in 2020 to the highest annual incidence in the observed study period, increasing to 673 elbow dislocations in 2020 from a mean of 406 annual elbow dislocations in previous years. A similar trend was seen in roller skating/skateboarding, with a 46% increase in elbow dislocations in 2020. The soccer and skating/skateboarding trends suggest that patients may have gravitated towards sports classically played outdoors in an environment where social distancing was possible. With regards to incident locale, the number of dislocations occurring at schools in 2020 decreased to only 3% (n = 6), representing a third of injury proportions from previous years, although the number of dislocations at unknown or unrecorded locations that year also increased from a mean of 23.3% to 33.6%.

**Table 2:** Comparison of Sports-Related Injuries during the COVID-19 Pandemic

Sport/Activity	Mean Elbow Dislocations prior to the COVID-19 Pandemic, 2011-2019, mean (SD)	Elbow Dislocations during the COVID-19 Pandemic, 2020, n	% Change	
				Football	709 (185)	419	- 41%	
Wrestling	707 (193)	257	- 64%	
Basketball	433 (85)	226	- 48%	
Bicycle	428 (209)	343	- 20%	
Soccer	406 (161)	673	66%	
Roller Skating/Skateboarding	397 (195)	581	46%	
Gymnastics	337 (171)	127	- 62%	
Cheerleading	168 (100)	86	- 49%	
Trampoline	166 (92)	19	- 88%	

## Discussion

This study provides a detailed assessment of epidemiologic trends specific to primary, simple elbow dislocations across the US. Utilization of the NEISS database in combination with recent US census data serves to reflect the most up-to-date trends and facilitates a unique understanding of the societal burden of elbow dislocations given that all included patients presented to an ED and received resources from this healthcare system. Ultimately, this study demonstrates that the incidence of elbow dislocations is closely linked to patient age and sex demographics as well as the implicated mechanism of injury, findings that enable a better understanding of these injury trends and offer insight into injury prevention.

Over the course of our study from 2011 through 2020, a national estimate of 83,994 elbow dislocations occurred among a cumulative annual population of over 2 trillion people, generating an incidence rate of 2.98 elbow dislocations per 100,000 persons. To our knowledge, the incidence of simple elbow dislocations has only been explored in three studies since 1980, with the most recent one published in 2015 [2,15,16]. In these three studies, incidence rates ranged from 2.65 to 5.21 dislocations per 100,000 people among populations in Sweden, Canada, and the US, with variability of dislocation rates likely attributable to differences in inclusion criteria. While our estimated incidence rate of 2.98 dislocations per 100,000 persons falls within this range, it is lower than previously reported by a prior exploration of NEISS data that demonstrated 5.21 simple elbow dislocations in patients over 10 per 100,000 people between 2002 - 2006 [2]. Still, many trends from this historical analysis were maintained in the present study: for example, a strong overall male predominance with the highest incidence among males 10 - 19 years, the increasing incidence in females with age, and the commonality of football and wrestling-related injuries (22% and 12%, respectively) [2]. One interesting sports-related difference was the higher frequency of skating injuries from 2002 - 2006, which were more common than wrestling injuries and accounted for 12% of dislocations in this time period rather than 9% [2].

With regards to patient demographics, our findings align with trends observed in previous literature: after a dramatic peak in earlier years, the incidence of simple elbow dislocations among males trended downwards [2,15,16]. Our data suggests that this trend may not be entirely due to athletic activity, with similar rates of elbow dislocations in male patients under 40 attributable to both sporting and non-sporting mechanisms (3.87 versus 2.18 events per 100,000 person-years, respectively). However, a very different trend was noted among female patients, where the incidence of elbow dislocations declined briefly in patients in their 30s and 40s before increasing to a consistently elevated rate. Mayne et al. [16] noted a similar increase in rates of elbow dislocations among Canadian women in the middle of life, which they attributed to an increased risk of falls and injury compared to men later in life, but there is no clear consensus on why the incidence rate ratio between men and women shifts so drastically in patients over 50.

Sporting activity accounted for nearly half of all isolated elbow dislocations (47%, national estimate n = 45,902), a finding that closely reflects historically reported mechanisms in the NEISS database from over a decade prior [2]. Among individual sports, football and wrestling had the highest incidence of injury, comprising a respective 15% (national estimate n = 6,797) and 14% (national estimate n = 6,627) of all sports injuries, with no other sports accounting for over 9% of these injuries. Elbow dislocations are classically caused by axial loading secondary to a fall; however, a study on elbow dislocations in NCAA athletes demonstrated that over 30% of injuries in both football and wrestling occurred secondary to person-to-person contact [17]. Subsequently, the higher rates of dislocations observed in sports like football and wrestling may be explained by the additional risk of injury with contact sports [17,18].

Data collected in 2020 demonstrate injury trends in the setting of the COVID-19 pandemic. While we observed the lowest incident rate in the past decade during this year with 2.21 dislocations per 100,000 persons, a rate that is much lower than the mean annual dislocation rate of 2.98 dislocations per 100,000 persons, rates of injuries reported in the literature are more variable. Some studies note increases in injuries like elbow fractures while others describe decreases in upper extremity injury rates compared to previous years [19-24]. Rates of injuries occurring during sporting activities in both males and females ages 10 - 39 were noted to be lower in 2020 as compared to previous means, and the sports often implicated in elbow dislocations transitioned from contact and indoor sports to soccer. With regards to location, the substantial decrease in injuries occurring at schools (3.8% in 2020 versus 9.4% mean from 2011 - 2019) likely reflects the prolonged school closures and transition to remote learning in addition to the athletic cancellations and restrictions on sporting activities and competitions during the pandemic even after schools opened. These findings contribute to an improved understanding of the effects of COVID-19 on injury rates, especially as they pertain to athletes, student-athletes, and different patient demographics.

Limitations

There are several limitations to this study. Firstly, NEISS data is collected by coders, introducing the likelihood for human error. Data were also limited by NEISS collection parameters as well as historical population statistics. While census data were available from 2011 through 2019, census projections are periodic with the most recent one from 2017; subsequently, the 2017 projections were used for the 2020 population data given that the 2020-specific data will unfortunately not be obtainable until 2031. Finally, data in the NEISS is specific to EDs and does not reflect elbow dislocations that presented primarily to urgent care centers or orthopaedic clinics, which may underestimate the overall incidence of these injuries. 

## Conclusions

Elbow dislocations are relatively common, with thousands of cases across the US annually. There are predictable trends regarding patient age and gender demographics, with decreasing incidence with age among men and increasing incidence in women over 40. Sports-related mechanisms are common, especially among contact sports and younger patients. The COVID-19 pandemic appears to impact both sports-related and epidemiologic injury patterns. Ultimately, a detailed and current understanding of population-level risk for elbow dislocations enables orthopaedic surgeons to better understand these injury trends, and educate patient populations on a broader level.
